# The association between oxidative balance score and sleep duration: a mediation analysis of a cross-sectional study

**DOI:** 10.3389/fnut.2024.1423424

**Published:** 2024-11-11

**Authors:** Guihua Hao, Xiaomei Zhao, Weiwei Fu, Yiwen Wu, Jingjing Dai, Yifeng Qian, Tian Xie, Lili Hou, Wentao Shi

**Affiliations:** ^1^Department of Nursing, Shanghai Ninth People’s Hospital, School of Medicine, Shanghai Jiaotong University, Shanghai, China; ^2^Shanghai Jiao Tong University School of Nursing, Shanghai, China; ^3^Department of Gastroenterology, Peking University Third Hospital, Beijing, China; ^4^Department of Oral and Craniomaxillofacial Surgery, Shanghai Ninth People’s Hospital, College of Stomatology, Shanghai Jiao Tong University School of Medicine, Shanghai, China; ^5^Department of General Surgery, Shanghai Ninth People’s Hospital, Shanghai Jiao Tong University School of Medicine, Shanghai, China; ^6^Clinical Research Unit, Shanghai Ninth People’s Hospital, School of Medicine, Shanghai Jiaotong University, Shanghai, China

**Keywords:** OBS, sleep duration, inflammation, oxidative stress, NHANES, mediation analysis

## Abstract

**Study objectives:**

The Oxidative Balance Score (OBS), which reflects overall oxidation through diet and lifestyle, has been linked to sleep, but few studies have clarified this relationship. We investigated the association between OBS and sleep duration, and whether oxidative stress (OS) and inflammation mediate the underlying mechanisms.

**Methods:**

Data were obtained from the National Health and Nutrition Examination Survey spanning the years 2007 to 2018. Multivariable logistic regression analyses were used to evaluate the association between OBS and the risk of sleep duration. Mediation analyses were conducted to investigate the role of OS and inflammatory markers.

**Results:**

A significant negative association was found between OBS and sleep duration (*p* < 0.01). Meanwhile, compared to participants in OBS tertile 1, the ORs (95% CIs) of incident short sleep duration were 0.78 (0.72–0.86) and 0.72 (0.67–0.79) (both *p* < 0.01) for OBS tertile 2 and 3, respectively. And the ORs (95% CIs) of incident long sleep duration were 0.83 (0.73, 0.95) and 0.66 (0.57, 0.75) (both *p* < 0.01) for OBS tertiles 2 and 3 after adjustment for multivariate variables. A linear relationship between OBS and short/long sleep duration (*p* for non-linearity = 0.69/0.94, both *p* < 0.01) were revealed. Mediation analysis showed absolute neutrophil count, serum total bilirubin mediated the association between OBS and short/long sleep duration with 5.72, 13.41% proportion of mediation, respectively (both *p* < 0.001).

**Conclusion:**

OBS is negatively associated with sleep duration. OS and inflammatory biomarkers mediate the relationship.

## 1 Introduction

Sleep plays a fundamental role in promoting physical and mental health by allowing the body to recover, repair and grow, and is a multifaceted phenomenon that can be characterized by different dimensions such as duration, timing, efficiency and regularity. Among these dimensions, sleep duration has been the most extensively studied (1). In 2020, 33.2% of adults reported sleeping for less than 7 h per day (2). The American Academy of Sleep Medicine (AASM) and the Sleep Research Society (SRS) have reached a consensus that adults should aim for 7–9 h of sleep per night to achieve optimal health (3, 4). Insufficient sleep has been linked to negative social and health outcomes in the general population, including cancer, cardiovascular disease, anxiety, and all-cause mortality (5, 6). In addition, insufficient sleep can impose an economic burden on society (7). In 2017, the estimated annual cost of insufficient sleep in the United States was between $280 billion and $411 billion (8). Given the significant negative social and personal consequences of insufficient sleep, it is important to identify risk factors and understand potential mechanisms for sleep duration.

Several factors, including depression, daily behaviors, and environmental conditions significantly impact sleep (9). Recent studies have strongly suggested that sleep duration appears to be associated with higher levels of oxidative stress (OS), as expressed by higher levels of serum pro-oxidant/antioxidant balance (10). In fact, previous study has shown that OS affects brain structures involved in the sleep–wake cycle, which can lead to sleep disorders (11). Several studies suggest that OS is the underlying pathophysiological mechanism for developing cardiovascular and neurobehavioral complications in obstructive sleep apnea (12). In addition, animal models have provided important evidence that prolonged wakefulness induces chronic oxidative stress, leading to a failure of antioxidant mechanisms (13). Considering the bidirectional relationship between sleep and oxidative stress, accurately assessing the level of OS is essential for maintaining sleep health. In light of this, we used the Oxidative Balance Score (OBS) as a valid tool to assess OS in our study. OBS was calculated to reflect the overall exposure to both pro-oxidants and antioxidants in diet and lifestyle, with higher scores indicating a predominance of antioxidant over pro-oxidant exposure (14). Numerous studies have demonstrated a significantly negative correlation between OBS and various health conditions, including cancer, hypertension and mortality (15). Based on the results of previous studies, there may be a link between OBS and sleep duration.

Investigating the pathways that may be involved in OBS effects on sleep duration is of increasing interest. Several studies have strongly suggested that abnormal inflammatory states may be an under-recognized factor in sleep, as indicated by increased production of pro-inflammatory cytokines and oxidative biomarkers (16). Moreover, OS and inflammatory processes are positively linked to abnormal sleep (17). A number of studies have shown that OBS can serve as a biomarker for both OS and inflammation (18). A study by Lee et al. indicated that higher levels of OBS were associated with lower levels of inflammatory markers, including C-reactive protein and white blood cell (WBC) count (19). Based on these studies, we hypothesized that low OBS may result in increased markers of OS and inflammation, which may subsequently lead to abnormal sleep duration. However, there is a lack of research examining whether the effects of OBS on sleep duration is mediated by OS and inflammatory markers.

Therefore, to address this knowledge gap, we used recent cross-sectional data from NHANES (*n* = 15,591) to examine the association between OBS and sleep duration. Furthermore, we aimed to investigate whether the relationship between OBS and sleep duration was mediated by OS and inflammatory markers.

## 2 Methods

### 2.1 Study population

This study analyzed NHANES data (2007–2018). NHANES is an ongoing, cross-sectional, and nationally representative survey in the United States. It is a major program of the National Center for Health Statistics (NCHS) that evaluates the health and nutritional status of the non-institutionalized US civilian population. It was approved and sponsored by the Centers for Disease Control and Prevention (CDC). The survey collected data biennially using a complex, multistage probability sampling procedure. NCHS-provided survey weights account for differential selection and nonresponse probabilities, allowing for the generation of nationally representative estimates. NHANES employs a computer-assisted personal interviewing (CAPI) system to conduct household interviews. This involved face-to-face interviews at participants’ residences, followed by a physical examination at a mobile examination center (MEC), where blood and urine samples were obtained. The NHANES was reviewed and approved by the NCHS Research Ethics Review Board, and all participants provided informed consent. The data from NHANES is available to the public. Additional information on the collection and analysis of the survey data can be found elsewhere.

**Figure 1:**
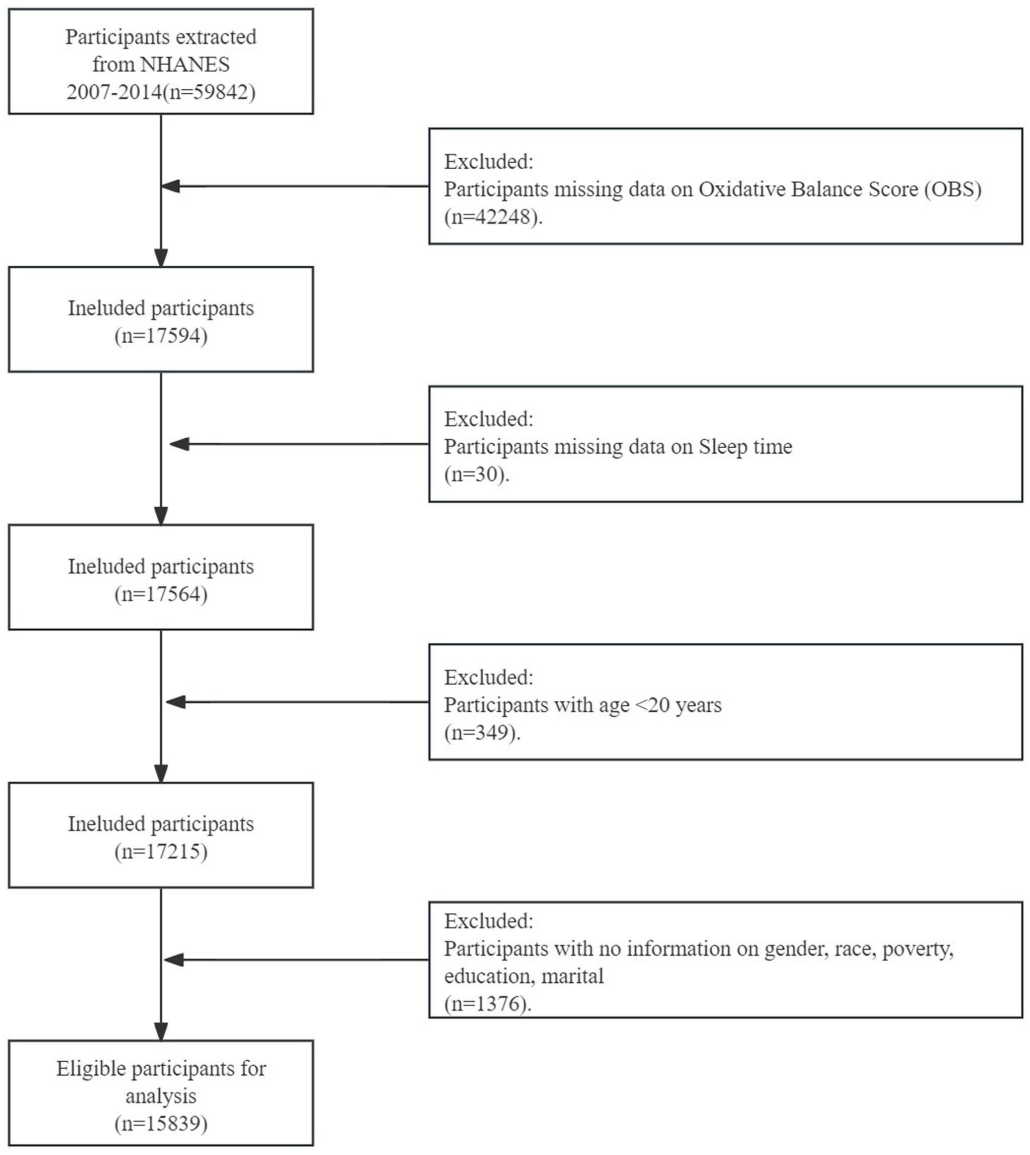
Flowchart of the sample selection from NHANES.

The cycles involved a total of 59,842 participants. 42,057 participants who lacked information on OBS components or sleep duration were excluded. Additionally, individuals were excluded if (1) they were less than 18 years old (*n* = 519), and (2) they lacked data on covariates or biomarkers (*n* = 1,675). Ultimately, a total of 15,591 participants were included (Figure 1).

### 2.2 Oxidative balance scores

The OBS for each participant was calculated using the method described in the relevant literature (5). This method selected 16 dietary components and four lifestyle components on the basis of prior knowledge of their association with oxidative stress. The components of OBS have been classified into four groups: (1) dietary antioxidants (fiber, *β*-carotene, riboflavin, niacin, vitamin B_6_, total folate, vitamin B_12_, vitamin C, vitamin E, calcium, magnesium, zinc, copper, and selenium), (2) dietary pro-oxidants (total fat and iron), (3) lifestyle antioxidants (physical activity), and (4) lifestyle pro-oxidants (alcohol, smoking, and body mass index [BMI]). All dietary components were categorized into tertiles based on their distribution in the study population. The scoring system assigned 0–2 points to participants in tertile 1 to 3 for dietary antioxidants; regarding dietary pro-oxidants, participants in tertile 1 were awarded 2 points, while those in tertile 3 received 0 points. The scores were then reversed accordingly. The scores for the lifestyle components have been calculated as follows: Physical activity was categorized as low (<150 min per week) = 0 points, moderate (150–300 min per week) =1 point, and high (>300 min per week) = 2 points (20). For alcohol, 0–2 points were assigned according to sex-specific levels of alcohol consumption. Smoking was assessed by measuring cotinine levels and scored on a scale of 0 to 2 for groups ranging from tertile 3 to tertile 1. BMI was calculated as weight (kg) divided by height squared (m^2^) and categorized as normal (< 25 kg/m^2^) = 2 points, overweight (25–29.9 kg/ m^2^) = 1 point, or obese (≥ 30 kg/m^2^) = 0 points. Finally, the OBS was calculated by summing the points assigned to each component. The scores ranged from 0 to 40 points. Furthermore, the dietary OBS was calculated by summing the scores of the 16 dietary components. Similarly, the lifestyle OBS was calculated by summing the scores of the four lifestyle components.

### 2.3 Assessment of sleep duration

Sleep duration was assessed by two questions that were consistent across the NHANES surveys: “How much sleep do you usually get at night on weekdays or workdays (hours)?” The response categories were represented by integers ranging from 1 to 12. A score of 12 indicates that the individual slept for 12 h or more. The optimal amount of sleep for adults is defined as 7–8 h per night by the American Academy of Sleep Medicine and Sleep Research Society (21). Thus, sleep duration was categorized into three groups: short (≤ 6 h), normal (7–8 h) and long (≥ 9 h).

### 2.4 Laboratory measures

Based on the potential association of oxidative stress, we included methylmalonic acid (MMA), bilirubin (BR), serum uric acid (SUA) and 25(OH)D_3_, and considering the potential inflammation association, we included neutrophil (NEU) and white blood cell (WBC). MMA was measured by gas chromatography–mass spectrometry (GC/MS) in 1999–2004 and by liquid chromatography-mass spectrometry (LC–MS/MS) in 2011–2014. Evaluation of MMA measurement by two methods showed excellent correlation and consistency (Deming regression, Bland–Altman analysis) for GC/MS and LC–MS/MS detection, supporting that MMA data measured by both protocols can be combined for analysis. BR (μmol/L) was measured using a timed endpoint diazo method on Beckman Synchron LX20. A colorimetric analysis at 520 nm at a fixed time interval was conducted by LX20. SUA was measured using a timed endpoint method on a Beckman UniCel DxC800 Synchron or a Beckman Synchron LX20 (Beckman Coulter, Inc., Brea, CA, USA). 25(OH)D_3_ were measured using high-performance liquid chromatography tandem mass spectrometry (HPLC-MS/MS). The reportable ranges of serum concentrations were 2.23–300 nmol/L for 25(OH)D_3_. WBC were measured using the complete blood count with the 5-part differential method during all four study periods. However, there was a change in the hematology analyzer used, with the Beckman Coulter MAXM being used in 2007–2012 and the Beckman Coulter DXH 800 being used in 2013–2018. NEU were determined by using the Beckman Coulter MAXM upon receipt of samples of MECs. The detailed method of obtaining variables mentioned above can be found at^1^.

### 2.5 Covariates

The study obtained sociodemographic information, including age, sex (female or male), race/ethnicity (Mexican-American, Non-Hispanic White, Non-Hispanic Black, and other), education (less than high school, high school, and college or higher), marital status (married or other), and family income-to-poverty ratio, and the combined diseases (included diabetes, hypertension, and tumor).

### 2.6 Statistical analysis

All analyses included sample weights to account for the complex sampling design of the NHANES. The baseline characteristics of the study participants were reported as mean and standard deviations (SD) for continuous variables and cases (n) and percentages (%) for categorical variables. OBS was modeled both as a continuous variable and tertiles, with tertile 1 as the reference group. Odds rations (ORs) and 95% confidence intervals (CIs) for the association between OBS and risk of abnormal sleep duration were estimated using multivariable logistic regression models. Three models were established: Model 1 was adjusted for no covariates; Model 2 was adjusted for sex, age, race, hypertension, diabetes, tumor, while Model 3 was adjusted for the variables in Model 2 plus education, marital status, and annual family income. Collinearity diagnosis results indicated that there is no multi-collinearity between the variables in Model 3. The dose–response association between OBS and risk of abnormal sleep duration was assessed using restricted cubic spline with a spline smoothing function. We further explored the association between dietary OBS/lifestyle OBS and abnormal sleep duration risk.

Mediation models were used to estimate the potential mediating effects of oxidative stress and inflammatory markers on the association between OBS and sleep duration. The individual mediating effect of oxidative stress and inflammation markers was estimated using the R package mediation. All mediation analyses were adjusted for age, sex, race/ethnicity, education, marital status, family income-to-poverty ration, health insurance, and chronic conditions. Indirect effect size (β_indirect_), direct effect size (β_direct_), total effect size (β_total_), proportion mediated (PM), and are presented in our results. We presented only biomarkers with PM > 4% in this study.

All statistical analyses were performed using SPSS for Windows, version 25.0 (SPSS) and *R* statistical package (*R* Project for Statistical Computing). Two-sided *p* values <0.05 were considered statistically significant.

## 3 Results

### 3.1 Basic characteristics of study participants

Among the 15,591 participants, 44.73% developed abnormal sleep duration. The mean age of the participants was 49.41 ± 17.36 years, with 8,173 (52.42%) males and 7,418 (47.58%) females. Of these participants, 7,572 (48.57%) were Non-Hispanic White, 8,743 (56.08%) had a college degree or above, 8,196 (52.57%) were married, the mean poverty-to-income ratio was 2.62 ± 1.64. Participants with higher OBS levels were more likely to be younger, male and have a higher proportion of non-black. They were also more often having a college degree or above, getting married and having a higher proportion of poverty-to-income ratio. Participants in OBS tertile 3 had the highest rate of achieving the recommended sleep duration. Notably, participants with higher OBS levels have lower MMA (Table 1).

**Table 1:** Baseline characteristics of participants according to the oxidative balance score’s tertile.

Characteristics	Total (*N* = 15,591)	T1 (<19) (*N* = 5,130)	T2 (19–26) (*N* = 4,812)	T3 (>26) (*N* = 5,649)	*p*
Age (years), mean (SD)	49.41 ± 17.36	51.09 ± 17.70	49.83 ± 17.32	47.53 ± 16.89	<0.01
Gender, *N* (%)					<0.01
Male	8,173 (52.42)	2,517 (49.06)	2,558 (53.16)	3,098 (54.84)	
Female	7,418 (47.58)	2,613 (50.94)	2,254 (46.84)	2,551 (45.16)	
Race/ethnicity, *N* (%)					<0.01
Mexican-American	2,143 (13.75)	655 (12.77)	656 (13.63)	832 (14.73)	
Non-Hispanic White	7,572 (48.57)	2,189 (42.67)	2,378 (49.42)	3,005 (53.20)	
Non-Hispanic Black	3,042 (19.51)	1,406 (27.41)	876 (18.20)	760 (13.45)	
Other-Race	2,834 (18.18)	880 (17.15)	902 (18.74)	1,052 (18.62)	
Education, *N* (%)					<0.01
Less than high school	3,292 (21.11)	1,468 (28.62)	1,012 (21.03)	812 (14.37)	
High School	3,549 (22.76)	1,373 (26.76)	1,101 (22.88)	1,075 (19.03)	
College or higher	8,743 (56.08)	2,286 (44.56)	2,696 (56.03)	3,761 (66.58)	
Marital status, *N* (%)					<0.01
Married	8,196 (52.57)	2,441 (47.58)	2,594 (53.91)	3,161 (55.96)	
Other	7,391 (47.41)	2,688 (52.40)	2,216 (46.05)	2,487 (44.03)	
PtI ratio, mean (SD)	2.62 ± 1.64	2.23 ± 1.54	2.67 ± 1.64	2.94 ± 1.67	<0.01
Sleep duration (hours), mean (SD)	6.98 ± 1.46	6.88 ± 1.64	7.02 ± 1.43	7.03 ± 1.29	<0.01
Sleep duration, *N* (%)					<0.01
Short	5,456 (34.99)	2044 (39.84)	1,622 (33.71)	1790 (31.69)	
Recommended	8,617 (55.27)	2,492 (48.58)	2,707 (56.26)	3,418 (60.51)	
Long	1,518 (9.74)	594 (11.58)	483 (10.04)	441 (7.81)	
Methylmalonic acid, median (Q1,Q3)	140.00 (108.00,185.00)	144.00 (110.00,194.00)	139.00 (106.50,186.00)	138.00 (107.00,178.00)	<0.01
TC (mg/dL), mean (SD)	193.74 ± 42.01	194.22 ± 43.08	194.12 ± 43.07	192.97 ± 40.08	0.62
TG (mg/dL), mean (SD)	127.26 ± 107.05	131.44 ± 102.39	130.65 ± 114.69	120.59 ± 104.20	
LDL-c (mg/dL), mean (SD)	114.41 ± 35.58	114.84 ± 36.90	114.70 ± 35.91	113.78 ± 34.05	0.74
HDL-c (mg/dL), mean (SD)	52.73 ± 16.30	51.73 ± 16.50	52.17 ± 15.97	54.11 ± 16.30	<0.01
HbA1c, %	5.73 ± 1.05	5.85 ± 1.15	5.75 ± 1.05	5.61 ± 0.92	<0.01
FBG,(mg/dL), mean (SD)	108.79 ± 35.32	112.41 ± 39.81	109.48 ± 36.42	104.89 ± 29.09	<0.01
Hypertension, *N* (%)					<0.01
Yes	5,600 (35.92)	2,138 (41.68)	1798 (37.36)	1,664 (29.46)	
No	9,991 (64.08)	2,992 (58.32)	3,014 (62.64)	3,985 (70.54)	
Diabetes, *N* (%)					<0.01
Yes	1990 (12.76)	852 (16.61)	658 (13.67)	480 (8.50)	
No	13,601 (87.24)	4,278 (83.39)	4,154 (86.33)	5,169 (91.50)	
Tumor, *N* (%)					0.97
Yes	1,585 (10.17)	518 (10.10)	489 (10.16)	578 (10.23)	
No	14,006 (89.83)	4,612 (89.90)	4,323 (89.84)	5,071 (89.77)	

PtI ratio, Poverty-to-income ratio; TC, Total Cholesterol; TG, triglyceride; LDL-c, Low density lipoprotein cholesterol; HDL-c, High-density lipoprotein cholesterol; HbA1c, Glycosylated hemoglobin; FBG, Fasting blood glucose.

### 3.2 Association between OBS and risk of abnormal sleep duration

**Figure 2:**
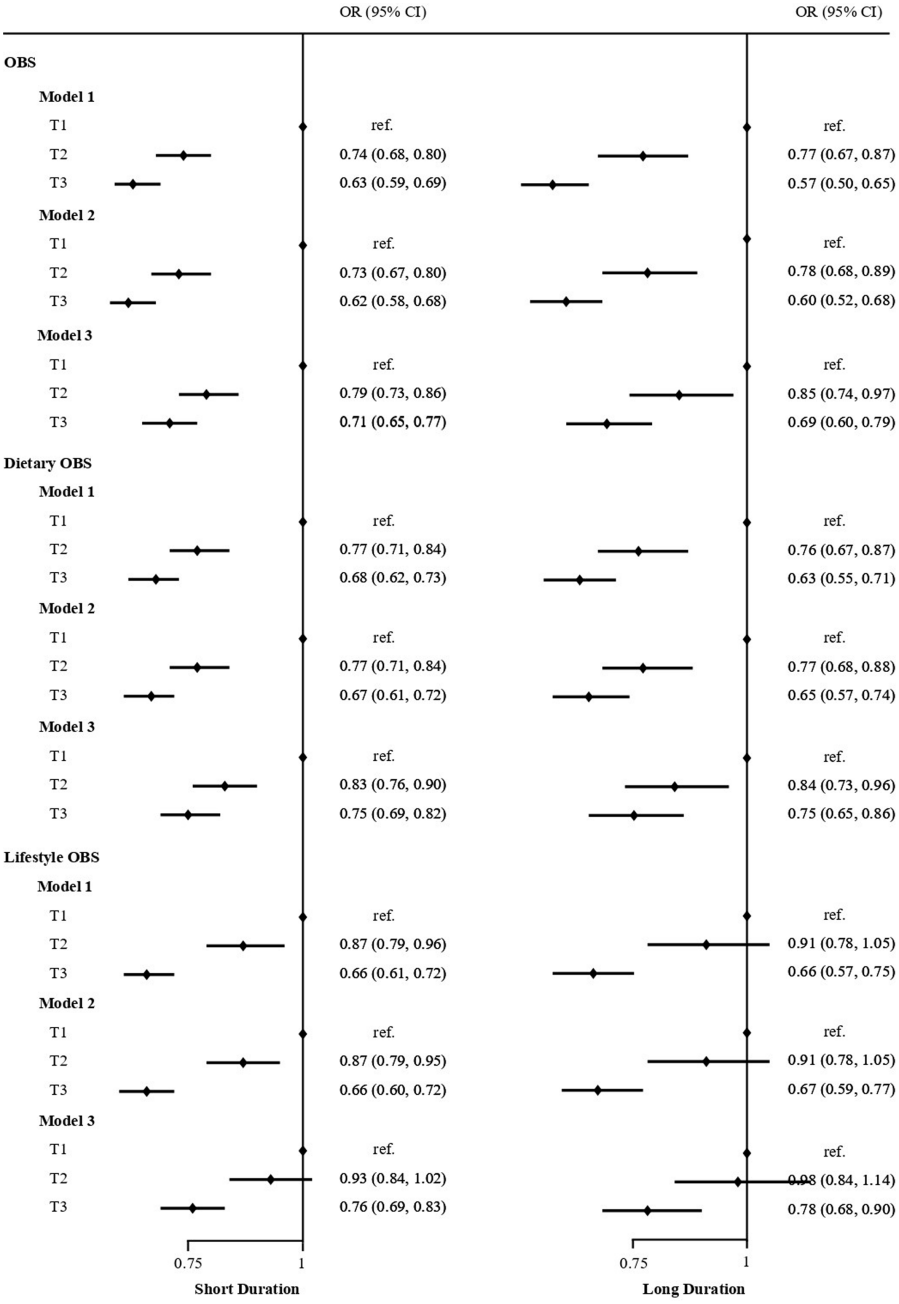
Logistic regression analyses of the association between OBS, Dietary OBS, Lifestyle OBS and sleep duration in US adult population. Adjust for: Model 1, no adjusted; Model 2, sex, age; Model 3, Model 2 plus race, education, marital status, and annual family income.

Figure 2 displayed the association between OBS and sleep duration based on logistic regression. The results indicate that individuals in the highest OBS tertile had a lower odds of short/long sleep duration. And the protective effect tended to increase with higher OBS values. Compared with OBS tertile 1, the ORs (95% CIs) of incident short sleep duration were 0.78 (0.72–0.86) and 0.72 (0.67–0.79) (both *p* < 0.01) for OBS tertile 2 and 3, respectively. And the ORs (95% CIs) of incident long sleep duration were 0.83 (0.73, 0.95) and 0.66 (0.57, 0.75) (both *p* < 0.01) for OBS tertile 2 and 3 after adjusting for multivariate variables in model 3. Stratified analyses by age, gender, and educational level yielded results consistent with those previously reported (Table 2).

**Table 2:** Crude and adjusted models showing the relationship between OBS and sleep duration in US adult population.

			Model 1				Model 2				Model 3			
			Short duration		Long duration		Short duration		Long duration		Short duration		Long duration	
		*N*	OR (95% CI)	*p*	OR (95% CI)	*p*	OR (95% CI)	*p*	OR (95% CI)	*p*	OR (95% CI)	*p*	OR (95% CI)	*p*
Gender														
Male	T1	2,517	Ref		Ref		Ref		Ref		Ref		Ref	
	T2	2,558	0.73 (0.67–0.80)	<0.01	0.75 (0.66–0.85)	<0.01	0.75 (0.68–0.81)	<0.01	0.77 (0.67–0.88)	<0.01	0.78 (0.72–0.86)	<0.01	0.83 (0.73–0.95)	<0.01
	T3	3,098	0.64 (0.59–0.69)	<0.01	0.54 (0.47–0.62)	<0.01	0.67 (0.61–0.72)	<0.01	0.58 (0.50–0.66)	<0.01	0.72 (0.67–0.79)	<0.01	0.66 (0.57–0.75)	<0.01
Female	T1	2,613	Ref		Ref		Ref		Ref		Ref		Ref	
	T2	2,254	0.73 (0.67–0.80)	<0.01	0.75 (0.66–0.85)	<0.01	0.75 (0.68–0.81)	<0.01	0.77 (0.67–0.88)	<0.01	0.78 (0.72–0.86)	<0.01	0.83 (0.73–0.95)	<0.01
	T3	2,551	0.64 (0.59–0.69)	<0.01	0.54 (0.47–0.62)	<0.01	0.67 (0.61–0.72)	<0.01	0.58 (0.50–0.66)	<0.01	0.72 (0.67–0.79)	<0.01	0.66 (0.57–0.75)	<0.01
Age														
<50	T1	2,338	Ref		Ref		Ref		Ref		Ref		Ref	
	T2	2,420	0.73 (0.67–0.80)	<0.01	0.75 (0.66–0.85)	<0.01	0.75 (0.68–0.81)	<0.01	0.77 (0.67–0.88)	<0.01	0.78 (0.72–0.86)	<0.01	0.83 (0.73–0.95)	<0.01
	T3	3,144	0.64 (0.59–0.69)	<0.01	0.54 (0.47–0.62)	<0.01	0.67 (0.61–0.72)	<0.01	0.58 (0.50–0.66)	<0.01	0.72 (0.67–0.79)	<0.01	0.66 (0.57–0.75)	<0.01
> = 50	T1	2,792	Ref		Ref		Ref		Ref		Ref		Ref	
	T2	2,392	0.73 (0.67–0.80)	<0.01	0.75 (0.66–0.85)	<0.01	0.75 (0.68–0.81)	<0.01	0.77 (0.67–0.88)	<0.01	0.78 (0.72–0.86)	<0.01	0.83 (0.73–0.95)	<0.01
	T3	2,505	0.64 (0.59–0.69)	<0.01	0.54 (0.47–0.62)	<0.01	0.67 (0.61–0.72)	<0.01	0.58 (0.50–0.66)	<0.01	0.72 (0.67–0.79)	<0.01	0.66 (0.57–0.75)	<0.01
Education														
Less than College	T1	2,841	Ref		Ref		Ref		Ref		Ref		Ref	
	T2	2,113	0.73 (0.67–0.80)	<0.01	0.75 (0.66–0.85)	<0.01	0.75 (0.68–0.81)	<0.01	0.77 (0.67–0.88)	<0.01	0.78 (0.72–0.86)	<0.01	0.83 (0.73–0.95)	<0.01
	T3	1887	0.64 (0.59–0.69)	<0.01	0.54 (0.47–0.62)	<0.01	0.67 (0.61–0.72)	<0.01	0.58 (0.50–0.66)	<0.01	0.72 (0.67–0.79)	<0.01	0.66 (0.57–0.75)	<0.01
College or higher	T1	2,286	Ref		Ref		Ref		Ref		Ref		Ref	
	T2	2,696	0.73 (0.67–0.80)	<0.01	0.75 (0.66–0.85)	<0.01	0.75 (0.68–0.81)	<0.0001	0.77 (0.67–0.88)	<0.01	0.78 (0.72–0.86)	<0.01	0.83 (0.73–0.95)	<0.01
	T3	3,761	0.64 (0.59–0.69)	<0.01	0.54 (0.47–0.62)	<0.01	0.67 (0.61–0.72)	<0.0001	0.58 (0.50–0.66)	<0.01	0.72 (0.67–0.79)	<0.01	0.66 (0.57–0.75)	<0.01

Model 1, no adjusted. Model2, Adjust for sex, age, race, hypertension, diabetes, tumor. Model3, Adjust for the variables in Model 2 plus education, marital status, and annual family income. OR (95% CI) odds ratio, 95 percent confidence interval. Recommended sleep duration is used as a reference category for sleep duration.

In the subsets analysis of OBS, participants in the highest tertile of dietary OBS were found to have lower odds of short sleep duration (OR: 0.76; 95% CI: 0.69–0.82; *p* < 0.001) and long sleep duration (OR: 0.72; 95% CI: 0.63–0.82; *p* < 0.001) after adjusting for all covariates. Similarly, participants in the highest tertile of lifestyle OBS had lower odds of short sleep duration (OR: 0.80; 95% CI: 0.73–0.88; *p* < 0.001) and long sleep duration (OR: 0.82; 95% CI: 0.71–0.95; *p* < 0.001; Figure 2; Supplementary Table S1).

**Figure 3:**
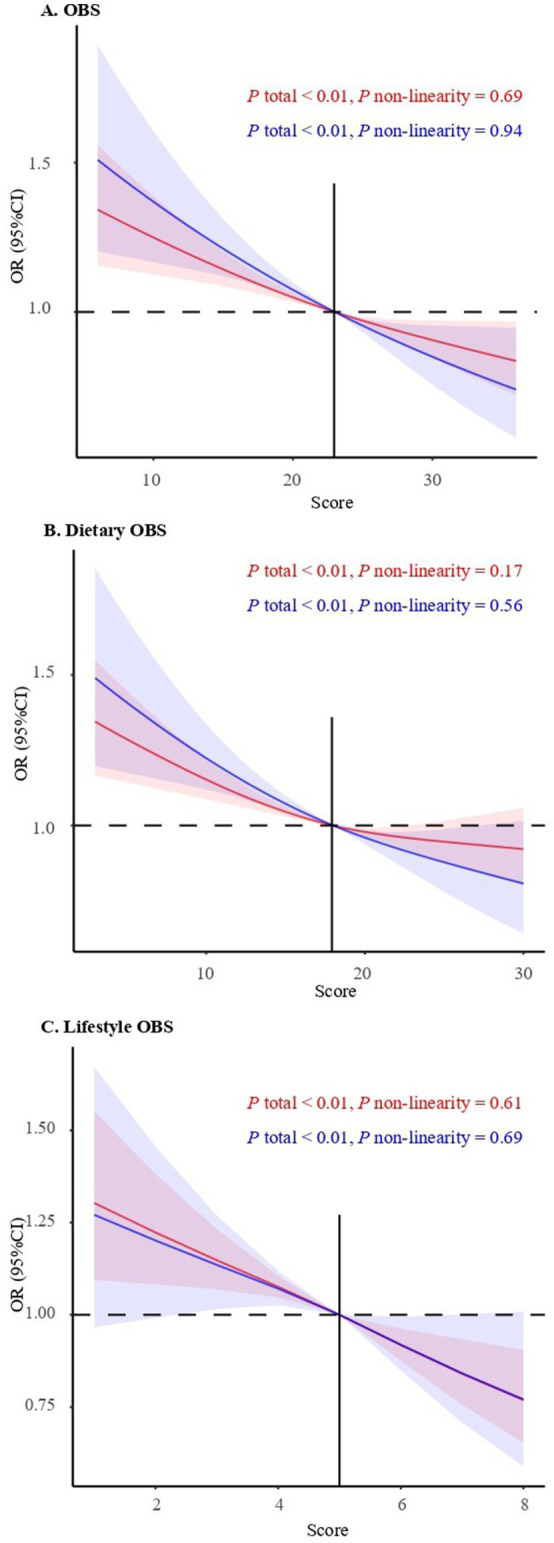
Dose–response associations between OBS, Dietary OBS, Lifestyle OBS and short/long sleep duration in model 3. The red line represents short sleep, while the blue line represents long sleep. The solid black lines correspond to the central estimates, and the gray-shaded regions indicate the 95% confidence intervals.

Dose response presents a linear trend in the association between OBS and short/long sleep duration (*p* for non-linearity = 0.69, *p* for non-linearity = 0.94, both *p* < 0.01). The cut-off value of the OBS that cause abnormal sleep duration were 22.95. Similarly, the dietary OBS, lifestyle OBS and short/long sleep duration also presents a linear trend (*p* for non-linearity = 0.17, *p* for non-linearity = 0.56; *p* for non-linearity = 0.61, *p* for non-linearity = 0.69, both *p* < 0.01). The cut-off value of the dietary OBS, lifestyle OBS that cause abnormal sleep duration were 17.92 and 5.00, respectively, (Figure 3).

### 3.3 Mediation analyses with separate mediators between OBS and sleep duration

Mediation analyses showed that NEU, WBC and the 25(OH)D_3_ had significant mediating effects on the association between OBS and short sleep duration, with 5.03, 5.72 and 4.12% proportion of mediation respectively, while the IDE was 1.001 (1.001–1.002), 1.002 (1.001–1.002) and 1.001 (1.000–1.002), (all *p* < 0.001). Furthermore, the mediation analyses showed that NEU, WBC counts, MMA, BR and SUA had significant mediating effects on the association between OBS and long sleep duration, with 9.98, 8.65, 4.99, 13.41 and 8.61% proportion of mediation respectively, while the IDE was 1.003 (1.002–1.004), 1.002 (1.001–1.003), 1.001 (1.000–1.002), 1.003 (1.002–1.005) and 1.002 (1.001–1.003), (all *p* < 0.001; Table 3).

**Table 3:** Mediation analyses with separate mediators between OBS and sleep duration in US adult population, NHANES 2007–2018.

Mediators	IDE	DE	TE	PM
	β_indirect (95% CI)_	β_direct (95% CI)_	β_total (95% CI)_	%
Short duration				
NEU	1.001 (1.001–1.002)	1.029 (1.022–1.035)	1.030 (1.024–1.036)	5.03
WBC	1.002 (1.001–1.002)	1.028 (1.022–1.035)	1.030 (1.024–1.036)	5.72
25(OH)D_3_	1.001 (1.000–1.002)	1.029 (1.023–1.035)	1.030 (1.024–1.036)	4.12
Long duration				
NEU	1.003 (1.002–1.004)	1.024 (1.014–1.034)	1.026 (1.016–1.037)	9.98
WBC	1.002 (1.001–1.003)	1.024 (1.014–1.034)	1.026 (1.016–1.036)	8.65
BR	1.003 (1.002–1.005)	1.023 (1.013–1.033)	1.026 (1.016–1.037)	13.41
SUA	1.002 (1.001–1.003)	1.024 (1.013–1.034)	1.026 (1.016–1.036)	8.61
MMA	1.001 (1.000–1.002)	1.015 (1.002–1.029)	1.016 (1.003–1.030)	4.99

NEU, absolute neutrophil count; WBC, white blood cell; 25(OH)D3, 25-hydroxyvitamin D_3_; BR, serum total bilirubin; SUA, serum uric acid; MMA, methylmalonic acid. IDE, Indirect Effect; DE, Direct Effect; TE, Total Effect; PM, Percentage Mediated.

## 4 Discussion

In this study, we examined the correlation between OBS and sleep duration in a large, nationally representative sample of US adults. Our main findings indicate a strong association between higher OBS and a lower risk of short/long sleep duration. Participants were more inclined to have a recommended sleep duration when OBS > 22, dietary OBS > 17, or lifestyle OBS > 5, suggesting that an antioxidant diet and a healthy lifestyle could be beneficial in improving sleep. Furthermore, oxidative stress (MMA, BR, SUA and 25(OH)D_3_) and inflammatory biomarkers (NEU, WBC) were founded to mediate the relationship between OBS and sleep duration. Our study is the first to investigate the relationship between OBS and sleep duration with recommended OBS score for dietary OBS, lifestyle OBS and total OBS, which may provide a new research basis for exploring the relationship between sleep, oxidative stress and inflammation, and a new guide for improving sleep in future studies.

The OBS is a composite score that reflects an individual’s antioxidant status by combining dietary and lifestyle factors (14). Several studies have evaluated the association between OS and sleep duration. A cohort study that identified a correlation between shorter sleep duration and increased serum pro-oxidant-antioxidant balance (22). Another study based on NHANES data reported that serum antioxidant status, particularly total carotenoids, can help poor sleepers improve their sleep duration due to their strong antioxidant properties (23). Our findings are consistent with those of previous studies, this study found that participants in OBS tertile 3 had lower odds of both short sleep duration (OR: 0.72; 95% CI: 0.67–0.79; *p* < 0.01) and long sleep duration (OR: 0.66; 95% CI: 0.57–0.75; *p* < 0.01) compared to those in tertile 1, after adjusting for all covariates. A linear relationship between OBS and the risk of short/long sleep duration was demonstrated using a restricted cubic spline. These findings indicate an important role for oxidative balance in sleep pathogenesis and progression.

Moreover, numerous studies have reported an inverse correlation between dietary antioxidants and sleep duration. In a cohort study of women in the UK, it was found that short sleepers consumed fewer fruits and vegetables (FV) and had lower levels of FV biomarkers, compared to the normal sleepers (24). Based on the NHANES data from 2005 to 2016, Ikonte et al. discovered that insufficient sleep was linked to a higher likelihood of nutrient inadequacy, particularly in antioxidant nutrients such as vitamins C and E, as well as lycopene (25). Additionally, several studies have examined the influence of lifestyle factors on sleep. A meta-analysis of daytime movement behaviors, found that a higher proportion of physical activity (PA) during the day is associated with a longer sleep duration (26). Moreover, middle-aged individuals who participated in moderate to vigorous physical activity were less likely to report a sleep disorder diagnosis than those who were less active (27). In our study, it was also found that both dietary OBS and lifestyle OBS were negatively and linearly associated with the risk of abnormal sleep duration. Participants in the highest tertile of dietary OBS were found to have lower odds of short sleep duration (OR: 0.76; 95% CI: 0.69–0.82) and long sleep duration (OR: 0.72; 95% CI: 0.63–0.82). Similarly, participants in the highest tertile of lifestyle OBS had lower odds of short sleep duration (OR: 0.80; 95% CI: 0.73–0.88) and long sleep duration (OR: 0.82; 95% CI: 0.71–0.95). Several studies have reported negative associations between OBS and inflammatory biomarkers. Sohouli et al. reported that individuals with higher OBS had reduced levels of F2-isoprostanes (FIP) and C-reactive protein (CRP) by 80 and 40%, respectively (28). However, previous studies were mostly observational, without exploring the underlying mechanisms.

Furthermore, the potential mediating effect of laboratory-based OS and inflammatory biomarkers between OBS and sleep duration was measured with mediation analyses in this study. The mediation analysis revealed that the association between OBS and short sleep duration may be mediated by NEU (5.03%), WBC (5.72%), and the 25(OH)D_3_ (4.12%). Additionally, NEU (9.98%), WBC (8.65%), MMA (4.99%), BR (13.41%) and SUA (8.61%) may mediate the association between OBS and long sleep duration. Previous study confirmed a bidirectional relationship between sleep and OS: OS triggers sleep, which then acts as an antioxidant for the brain (29). Changes in OS are positively related to loop gain and apnoea severity during non-rapid eye movement (NREM) sleep (30). Furthermore, the brain is more vulnerable to damage caused by increased ROS compared to other organs in the body. Studies have demonstrated that increased ROS production and depletion of antioxidant defenses are responsible for changes in brain structure (31). Additionally, a systematic review provides evidence for the antioxidant function of sleep in both brain and non-brain areas (32). Sleep is also associated with various inflammatory process, several inflammatory molecules and pathways can modulate sleep (33). Short sleep seems to be associated with an increase in inflammation and a higher risk of infection (34).

As previously mentioned, OS and inflammation are significant contribute to sleep disorders, including abnormal sleep duration. In fact, NEU, WBC count, MMA, 25(OH)D_3_, BR and SUA were associated with abnormal sleep duration in our study. It should be noted that inflammation can both increase and disrupt sleep duration and intensity (35). Inflammatory molecules in both central nervous system and periphery can alter sleep (33). Changes in hematology data such as WBC, NEU are common to virtually all inflammatory conditions, as well as sensitive indicators of inflammation (36). In recent years, WBC and NEU have received increasing attention as they are predictive of several disorders such as rest-activity circadian rhythm (37). Likewise, a cross-sectional study of US adults discovered that sedentary behavior was statistically associated with sleep disturbance, which was mediated by WBC and NEU (38). BR has been shown to have antioxidant, anti-inflammatory, and immunosuppressive effects (39). Several studies have reported the temporal association between BR and rapid-eye-movement (REM) sleep in the population and in different species (40). The hypothesis that BR plays a role in the regulation of REM sleep is based on the responses of BR to light (41). Due to the biological effects of vitamin D on various body systems, there has been a growing amount of interest in its potential role in sleep regulation. The majority of relevant epidemiological studies typically measure 25(OH) D_3_ to determine the body’s vitamin D levels. Koshi et al. discovered that individuals in the first quartile group with the lowest 25(OH)D_3_ concentrations were significantly more likely to have poor sleep quality in the adult population of Japan (42). SUA is the end product of purines and helps protect nerve cells through free radical scavenging and extracellular antioxidant activity (43). A recent study found that in patients with REM sleep behavior disorder, the positive relationship between the SUA and the functional connectivity of the substantia nigra with the left lingual gyrus was reduced, which was elucidated from a pathophysiological perspective (44). In addition, Li et al. found that SUA affect cognitive function in patients with idiopathic rapid eye movement sleep behavior disorder, which could contribute to its antioxidant and neuroprotective roles (45). MMA, a dicarboxylic acid, is primarily a by-product of propionate metabolism. It can be used as a diagnostic tool for hereditary MMA and to assess vitamin B12 status (46). It is noteworthy that there is increasing evidence of the key role of MMA in mitochondrial dysfunction and oxidative stress. According to Yuan et al., MMA can impact the central nervous system, resulting in structural changes or abnormal signals on brain MRI (47). Additionally, a study has revealed that low plasma MMA levels may be linked to white matter injury in mice experiencing long-term intermittent hypoxia, and potentially in those with obstructive sleep apnea (48). Consistent with these findings, we discovered that MMA mediates the association between OBS and long sleep duration. This can explain the relationship between oxidative stress and sleep at a mechanistic level. These available studies suggest that OS and inflammatory markers may act as mediators in the association between OBS and sleep duration.

This study has several strengths. Firstly, the individual’s antioxidant status was assessed using the OBS, a composite score that combines dietary and lifestyle factors. Previous studies on the relationship between diet and sleep have typically used the Dietary inflammatory index (DII) (49). However, it should be noted that the DII only responds to inflammatory states. In contrast, the OBS responds not only to inflammation, but also to oxidative stress. It assesses the impact of lifestyle on sleep in addition to diet, making this index a more reasonable guide for sleep studies. Secondly, we used a national population with a sample size that was sufficiently large. Thirdly, we evaluated the relationship between OBS and sleep duration, and also evaluated the relationship between dietary/lifestyle OBS and sleep duration, allowing a better evaluation of the role played by dietary and lifestyle habits on sleep duration. Finally, mediation analyses were used to explore the potential factors affecting OBS and sleep duration. This was done to better reveal the mechanisms underlying the relationship between OBS and sleep duration.

There are limitations to this study. Firstly, the duration of sleep is self-reported during the interview without any objective measurement, which may introduce information bias and contribute to the variability of the results, the analysis may have been affected by residual and unmeasured confounding as well. Secondly, we used the average of two 24-h recalls for estimation of dietary components, which may introduce bias. Thirdly, all measurements were taken only at baseline, but during long-term follow-up, participants’ lifestyles and diets may change over time. Finally, our study is a cross-sectional analysis and that it is difficult to draw conclusions about the direction of the effect, as reverse causation and residual confounding cannot be excluded. Further prospective randomized controlled research is required to confirm our findings.

## 5 Conclusion

The study revealed that higher OBS was linked to a lower likelihood of abnormal sleep duration. In addition, the association between OBS and sleep duration was found to be mediated by NEU count, WBC count, MMA, 25(OH)D_3_, BR and SUA, which indicated the mechanism of sleep duration involved in OS and inflammatory response. Future prospective and experimental studies are required to confirm this association and its underlying mechanisms.

## Data Availability

The original contributions presented in the study are included in the article/Supplementary material, further inquiries can be directed to the corresponding authors.

## Footnotes

^1^ www.cdc.gov/nchs/nhanes

## References

[1] Austin-Zimmerman I Levey DF Giannakopoulou O Deak JD Galimberti M Adhikari K. Genome-wide association studies and cross-population meta-analyses investigating short and long sleep duration. Nat Commun. (2023) 14:6059. doi: 10.1038/s41467-023-41249-y, PMID: 37770476 PMC10539313

[2] Pankowska MM Lu H Wheaton AG Liu Y Lee B Greenlund KJ. Prevalence and geographic patterns of self-reported short sleep duration among US adults. Prev Chronic Dis. (2023) 20:53. doi: 10.5888/pcd20.220400, PMID: 37384831 PMC10317035

[3] Watson NF Badr MS Belenky G. Joint consensus statement of the American Academy of sleep medicine and Sleep Research Society on the recommended amount of sleep for a healthy adult: methodology and discussion. J Clin Sleep Med. (2015) 11:931–52. doi: 10.5664/jcsm.4950, PMID: 26235159 PMC4513271

[4] Hirshkowitz M Whiton K Albert SM Alessi C Bruni O DonCarlos L. National sleep foundation’s updated sleep duration recommendations: final report sleep. Health. (2015) 1:233–43. doi: 10.1016/j.sleh.2015.10.004, PMID: 29073398

[5] Itani O Jike M Watanabe N Kaneita Y. Short sleep duration and health outcomes: a systematic review, meta-analysis, and meta-regression. Sleep Med. (2017) 32:246–56. doi: 10.1016/j.sleep.2016.08.006, PMID: 27743803

[6] Han H Wang Y Li T Feng C Kaliszewski C Su Y. Sleep duration and risks of incident cardiovascular disease and mortality among people with type 2 diabetes. Diabetes Care. (2023) 46:101–10. doi: 10.2337/dc22-1127, PMID: 36383480

[7] Glick DR Abariga SA Thomas I Shipper AG Gunia BC Grandner MA. Economic impact of insufficient and disturbed sleep in the workplace. Pharmacoeconomics. (2023) 41:771–85. doi: 10.1007/s40273-023-01249-8, PMID: 36933184

[8] Hafner M Stepanek M Taylor J van Stolk C van Stolk C. Why sleep matters-the economic costs of insufficient sleep: a cross-country comparative analysis. Rand Health Q. (2017) 6:11. PMID: 28983434 10.7249/rhq-06-04-11 PMC5627640

[9] Baranwal N Yu PK Siegel NS. Sleep physiology, pathophysiology, and sleep hygiene. Prog Cardiovasc Dis. (2023) 77:59–69. doi: 10.1016/j.pcad.2023.02.005, PMID: 36841492

[10] Liu J Wang W Wen Y. Association of dietary oxidative balance score and sleep duration with the risk of mortality: prospective study in a representative US population. Public Health Nutr. (2023) 26:2066–75. doi: 10.1017/S1368980023001155, PMID: 37309207 PMC10564614

[11] Yang Z Zhang X Li C Chi S Xie A. Molecular mechanisms underlying reciprocal interactions between sleep disorders and Parkinson’s disease. Front Neurosci. (2021) 14:592989. doi: 10.3389/fnins.2020.592989, PMID: 33642969 PMC7902929

[12] Gozal D Kheirandish-Gozal L. Cardiovascular morbidity in obstructive sleep apnea: oxidative stress, inflammation, and much more. Am Rev Respir Dis. (2008) 177:369–75. doi: 10.1164/rccm.200608-1190PP, PMID: 17975198 PMC2258438

[13] Atrooz F Salim S. Sleep deprivation, oxidative stress and inflammation. Adv Protein Chem Struct Biol. (2020) 119:309–36. doi: 10.1016/bs.apcsb.2019.03.001, PMID: 31997771

[14] Goodman M Bostick RM Dash C Terry P Flanders WD Mandel J. A summary measure of pro- and anti-oxidant exposures and risk of incident, sporadic, colorectal adenomas. Cancer Causes Control. (2008) 19:1051–64. doi: 10.1007/s10552-008-9169-y, PMID: 18543072

[15] Hernández-Ruiz Á García-Villanova B Guerra-Hernández E Amiano P Ruiz-Canela M Molina-Montes E. A review of a priori defined oxidative balance scores relative to their components and impact on health outcomes. Nutrients. (2019) 11:774. doi: 10.3390/nu11040774, PMID: 30987200 PMC6520884

[16] Zhang Y Zhao W Liu K Chen Z Fei Q Ahmad N. The causal associations of altered inflammatory proteins with sleep duration, insomnia and daytime sleepiness. Sleep. (2023) 46:zsad207. doi: 10.1093/sleep/zsad207, PMID: 37535878

[17] Almalki WH Ghoneim MM Alshehri S Imam SS Kazmi I Gupta G. Sepsis triggered oxidative stress-inflammatory axis: the pathobiology of reprogramming in the normal sleep-wake cycle. Mol Cell Biochem. (2022) 477:2203–11. doi: 10.1007/s11010-022-04432-1, PMID: 35451739

[18] Li H Song L Cen M Fu X Gao X Zuo Q. Oxidative balance scores and depressive symptoms: mediating effects of oxidative stress and inflammatory factors. J Affect Disord. (2023) 334:205–12. doi: 10.1016/j.jad.2023.04.134, PMID: 37149058

[19] Lee HS Park T. Pathway‐driven approaches of interaction between oxidative balance and genetic polymorphism on metabolic syndrome. Oxidative Med Cell Longev. (2017) 2017:6873197. doi: 10.1155/2017/6873197, PMID: 28191276 PMC5278231

[20] Piercy KL Troiano RP Ballard RM Carlson SA Fulton JE Galuska DA. The physical activity guidelines for Americans. JAMA. (2018) 320:2020–8. doi: 10.1001/jama.2018.14854, PMID: 30418471 PMC9582631

[21] Watson NF Badr MS Belenky G Bliwise DL Buxton OM Buysse D. Recommended amount of sleep for a healthy adult: a joint consensus statement of the American Academy of sleep medicine and Sleep Research Society. Sleep Adv. (2015) 38:843–4. doi: 10.5665/sleep.4716, PMID: 26039963 PMC4434546

[22] Darroudi S Eslamiyeh M Jaber al-Fayyadh KK Zamiri Bidary M Danesteh S Hassanzadeh Gouji A. Prognostic factors associated with sleep duration: serum pro-oxidant/antioxidant balance and superoxide dismutase 1 as oxidative stress markers and anxiety/depression. Int J Public Health. (2023) 68:1606014. doi: 10.3389/ijph.2023.1606014, PMID: 37744415 PMC10512420

[23] Deng MG Liu F Wang K Liang Y Nie JQ Liu J. Relationship between dietary carotenoid intake and sleep duration in American adults: a population-based study. Nutr J. (2023) 22:68. doi: 10.1186/s12937-023-00898-x, PMID: 38062512 PMC10704834

[24] Noorwali E Hardie L Cade J. Fruit and vegetable consumption and their polyphenol content are inversely associated with sleep duration: prospective associations from the UK women's cohort study. Nutrients. (2018) 10:1803. doi: 10.3390/nu10111803, PMID: 30463296 PMC6266198

[25] Ikonte CJ Mun JG Reider CA Grant RW Mitmesser SH. Micronutrient inadequacy in short sleep: analysis of the NHANES 2005-2016. Nutrients. (2019) 11:2335. doi: 10.3390/nu11102335, PMID: 31581561 PMC6835726

[26] Huang WY Ho RS Tremblay MS Wong SH. Relationships of physical activity and sedentary behaviour with the previous and subsequent nights' sleep in children and youth: a systematic review and meta-analysis. J Sleep Res. (2021) 30:e13378. doi: 10.1111/jsr.13378, PMID: 34235808

[27] Farnsworth JL Kim Y Kang M. Sleep disorders, physical activity, and sedentary behavior among U.S. adults: National Health and nutrition examination survey. J Phys Act Health. (2015) 12:1567–75. doi: 10.1123/jpah.2014-0251, PMID: 25710522

[28] Sohouli MH Rohani P Hosseinzadeh M Hekmatdoost A. Adherence to oxidative balance scores and lower odds of non-alcoholic fatty liver disease: a case-control study. Sci Rep. (2023) 13:6140. doi: 10.1038/s41598-023-33407-5, PMID: 37061551 PMC10105695

[29] Hill VM O’Connor RM Sissoko GB Irobunda IS Leong S Canman JC. A bidirectional relationship between sleep and oxidative stress in drosophila. PLoS Biol. (2018) 16:e2005206. doi: 10.1371/journal.pbio.2005206, PMID: 30001323 PMC6042693

[30] Panza GS Alex RM Yokhana SS Lee Pioszak DS Badr MS Mateika JH. Increased oxidative stress, loop gain and the arousal threshold are clinical predictors of increased apnea severity following exposure to intermittent hypoxia Nat Sci. Sleep. (2019) 11:265–79. doi: 10.2147/NSS.S228100 PMC6817348 31695534

[31] Bhatt S Nagappa AN Patil CR. Role of oxidative stress in depression. Drug Discov Today. (2020) 25:1270–6. doi: 10.1016/j.drudis.2020.05.001 32404275

[32] Villafuerte G Miguel-Puga A Murillo Rodríguez E Machado S Manjarrez E Arias-Carrión O. Sleep deprivation and oxidative stress in animal models: a systematic review. Oxidative Med Cell Longev. (2015) 2015:234952:1–15. doi: 10.1155/2015/234952 PMC4402503 25945148

[33] Zielinski MR Gibbons AJ. Neuroinflammation, sleep, and circadian rhythms. Front Cell Infect Microbiol. (2022) 12:853096. doi: 10.3389/fcimb.2022.853096 35392608 PMC8981587

[34] Akkaoui MA Palagini L Geoffroy PA. Sleep immune cross talk and insomnia. Adv Exp Med Biol. (2023) 1411:263–73. doi: 10.1007/978-981-19-7376-5_12, PMID: 36949314

[35] Besedovsky L Lange T Haack M. The sleep-immune crosstalk in health and disease. Physiol Rev. (2019) 99:1325–80. doi: 10.1152/physrev.00010.2018, PMID: 30920354 PMC6689741

[36] Germolec DR Shipkowski KA Frawley RP Evans E. Markers of inflammation. Methods Mol Biol. (2018) 1803:57–79. doi: 10.1007/978-1-4939-8549-4_5 29882133

[37] Xu Y Su S McCall WV. Blunted rest-activity rhythm is associated with increased white blood-cell-based inflammatory markers in adults: an analysis from NHANES 2011-2014. Chronobiol Int. (2022) 39:895–902. doi: 10.1080/07420528.2022.2048663, PMID: 35260021 PMC9117463

[38] You Y Chen Y Fang W. The association between sedentary behavior, exercise, and sleep disturbance: a mediation analysis of inflammatory biomarkers. Front Immunol. (2023) 13:1080782. doi: 10.3389/fimmu.2022.1080782 36713451 PMC9880546

[39] Vitek L Hinds TD Jr Stec DE Tiribelli C. The physiology of bilirubin: health and disease equilibrium. Trends Mol Med. (2023) 29:315–28. doi: 10.1016/j.molmed.2023.01.007, PMID: 36828710 PMC10023336

[40] Oren DA. Bilirubin, REM sleep, and phototransduction of environmental time cues. A hypothesis. Chronobiol Int. (1997) 14:319–29. doi: 10.3109/07420529709001423, PMID: 9167892

[41] Oren DA Desan PH Boutros N Anand A Charney DS. Effects of light on low nocturnal bilirubin in winter depression: a preliminary report. Biol Psychiatry. (2002) 51:422–5. doi: 10.1016/S0006-3223(01)01254-9 11904137

[42] Nakamura K Hui SP Ukawa S Okada E Nakagawa T Okabe H. Serum 25-hydroxyvitamin D3 levels and poor sleep quality in a Japanese population: the DOSANCO health study. Sleep Med. (2019) 57:135–40. doi: 10.1016/j.sleep.2019.01.046, PMID: 30981957

[43] Zhou C Gu M Yin L Yin W Liu J Zhu Y. Low serum uric acid levels may be a potential biomarker of poor sleep quality in patients with Parkinson's disease. Sleep Med. (2023) 105:9–13. doi: 10.1016/j.sleep.2023.03.011, PMID: 36934617

[44] Ellmore TM Suescun J Castriotta RJ Schiess MC. A study of the relationship between uric acid and substantia Nigra brain connectivity in patients with REM sleep behavior disorder and Parkinson's disease. Front Neurol. (2020) 11:815. doi: 10.3389/fneur.2020.00815, PMID: 32849245 PMC7419698

[45] Li X Jia S Zhou Z Jin Y Zhang X Hou C. Effect of serum uric acid on cognition in patients with idiopathic REM sleep behavior disorder. J Neural Transm - Parkinson's Dis Dement Sect. (2018) 125:1805–12. doi: 10.1007/s00702-018-1935-8, PMID: 30284075

[46] Gomes AP Ilter D Low V Endress JE Fernández-García J Rosenzweig A. Age-induced accumulation of methylmalonic acid promotes tumor progression. Nature. (2020) 585:283–7. doi: 10.1038/s41586-020-2630-0 32814897 PMC7785256

[47] Yuan Y Ma Y Wu Q Huo L Liu CF Liu X. Clinical and electroencephalogram characteristics of methylmalonic acidemia with MMACHC and MUT gene mutations. BMC Pediatr. (2024) 24:119. doi: 10.1186/s12887-024-04559-8, PMID: 38355526 PMC10865547

[48] Veasey SC Lear J Zhu Y Grinspan JB Hare DJ Wang SH. Long-term intermittent hypoxia elevates cobalt levels in the brain and injures white matter in adult mice. Sleep. (2013) 36:1471–81. doi: 10.5665/sleep.3038, PMID: 24082306 PMC3773196

[49] Wirth MD Liu J Wallace MK. Dietary inflammatory index and sleep quality and duration among pregnant women with overweight or obesity. Sleep. (2022) 45:zsac241. doi: 10.1093/sleep/zsac241, PMID: 36173829 PMC9742888

