# Corrigendum: The association between oxidative balance score and sleep duration: a mediation analysis of a cross-sectional study

**DOI:** 10.3389/fnut.2024.1537672

**Published:** 2024-12-16

**Authors:** Guihua Hao, Xiaomei Zhao, Weiwei Fu, Yiwen Wu, Jingjing Dai, Yifeng Qian, Tian Xie, Lili Hou, Wentao Shi

**Affiliations:** ^1^Department of Nursing, Shanghai Ninth People's Hospital, School of Medicine, Shanghai Jiaotong University, Shanghai, China; ^2^Shanghai Jiao Tong University School of Nursing, Shanghai, China; ^3^Department of Gastroenterology, Peking University Third Hospital, Beijing, China; ^4^Department of Oral and Craniomaxillofacial Surgery, Shanghai Ninth People's Hospital, College of Stomatology, Shanghai Jiao Tong University School of Medicine, Shanghai, China; ^5^Department of General Surgery, Shanghai Ninth People's Hospital, Shanghai Jiao Tong University School of Medicine, Shanghai, China; ^6^Clinical Research Unit, Shanghai Ninth People's Hospital, School of Medicine, Shanghai Jiaotong University, Shanghai, China

**Keywords:** OBS, sleep duration, inflammation, oxidative stress, NHANES, mediation analysis

In the published article, there was an error in the author list. The authors, Xiaomei Zhao and Weiwei Fu, were erroneously not indicated as sharing the position of first authorship with Guihua Hao. The corrected author list appears below.

Guihua Hao^1,2†^, Xiaomei Zhao^1†^, Weiwei Fu^3†^, Yiwen Wu^1^, Jingjing Dai^1^, Yifeng Qian^4^, Tian Xie^5*^, Lili Hou^1*^ and Wentao Shi^6*^. ^†^These authors have contributed equally to this work and share first authorship.

The authors apologize for this error and state that this does not change the scientific conclusions of the article in any way. The original article has been updated.

