# Supplementary material for: The association between oxidative balance score and sleep duration: a mediation analysis of a cross-sectional study
Source: Front Nutr. 2024 Nov 11;11:1423424. doi: 10.3389/fnut.2024.1423424 (PMC11586185; doi:10.3389/fnut.2024.1423424)
Supplement: Supplementary file 1 [file Table_1.DOCX]

**Table S1 Crude and adjusted models showing the relationship between OBS and sleep duration in US adult population**

|  |  | **Model1** | | **Model2** | | **Model3** | |
| --- | --- | --- | --- | --- | --- | --- | --- |
|  |  | **OR(95%CI)** | **P** | **OR(95%CI)** | **P** | **OR(95%CI)** | **P** |
| **Total OBS** | **Short duration** | 0.97(0.97-0.98) | <.01 | 0.97(0.97-0.98) | <.01 | 0.98(0.98-0.99) | <.01 |
|  | **Long duration** | 0.97(0.96-0.97) | <.01 | 0.97(0.96-0.98) | <.01 | 0.98(0.97-0.99) | <.01 |
| **Dietary OBS** | **Short duration** | 0.98(0.97-0.98) | <.01 | 0.98(0.97-0.98) | <.01 | 0.98(0.98-0.99) | <.01 |
|  | **Long duration** | 0.97(0.96-0.98) | <.01 | 0.97(0.96-0.98) | <.01 | 0.98(0.97-0.99) | <.01 |
| **Lifestyle OBS** | **Short duration** | 0.87(0.85-0.89) | <.01 | 0.87(0.85-0.89) | <.01 | 0.91(0.89-0.93) | <.01 |
|  | **Long duration** | 0.87(0.84-0.90) | <.01 | 0.88(0.84-0.91) | <.01 | 0.92(0.88-0.95) | <.01 |

Model 1: no adjusted. Model2: Adjust for sex, age, race, hypertension, diabetes, tumor. Model3: Adjust for the variables in Model 2 plus education, marital status, and annual family income. OR (95%CI) odds ratio, 95 percent confidence interval. Recommended sleep duration is used as a reference category for sleep duration.

**Table S2. The results of intermediary analysis.**

| **OS and inflammatory**  **biomarkers** | **short sleep duration** | | **long sleep duration** | |
| --- | --- | --- | --- | --- |
|  | **PM** | **IDE** | **PM** | **IDE** |
| SII | 1.33 | 1.0004(1.0001-1.0007) | 1.93 | 1.0005(1.000-1.001) |
| NLR | 0.63 | 1.0002(1.000-1.0004) | 1.53 | 1.0004(1.000-1.0008) |
| PLR | -0.06 | 1.000(0.9998-1.0001) | 0.12 | 1.000 (0.9998-1.0003) |
| LMR | 0.15 | 1.000 (0.9998-1.0003) | -1.588 | 0.9996(0.9991-1.0001) |
| Neutrophils percent | -0.04 | 1.000(0.9998-1.0002) | 0.61 | 1.0002(0.9998-1.0005) |
| Platelet count | 1.65 | 1.0005(1.0001-1.0008) | 2.38 | 1.0006(1.000-1.0012) |
| Lymphocyte number | 1.37 | 1.0004(1.000-1.0008) | 0.97 | 1.0002(1.000-1.0005) |
| Lymphocyte percent | -0.20 | 0.9999(0.9998-1.0001) | 0.42 | 1.0001(0.9997-1.0005) |
| Monocyte percent | 1.10 | 1.0003(1.0001-1.0006) | 0.06 | 1.000 (0.9996-1.0004) |
| Albumin-urine | 0.43 | 1.0001(0.9999-1.0004) | 1.15 | 1.0003(1.000-1.0006) |
| Albumin creatinine ratio | 0.31 | 1.0001(0.9999-1.0003) | 0.98 | 1.0003(1.000-1.0006) |
| GGT | 2.35 | 1.0007(1.0003-1.001) | 2.36 | 1.0006(1.0002-1.001) |
| 25OHD2+25OHD3 | -3.87 | 0.9989(0.9978-0.9999) | -3.79 | 0.9977(0.996-0.9993) |
| 25OHD2 | 2.11 | 1.0006(1.0003-1.001) | 2.42 | 1.0006(1.0002-1.0011) |

Abbreviations: SII, systemic immune-inflammation index; NLR, neutrophil-lymphocyte ratio; PLR, platelet to lymphocyte ratio; LMR, lymphocyte-to-monocyte ratio; GGT, Gamma glutamyl transferase.
